# Comparing Predictions of a PBPK Model for Cyclosporine With Drug Levels From Therapeutic Drug Monitoring

**DOI:** 10.3389/fphar.2021.630904

**Published:** 2021-05-14

**Authors:** Sonja E. Zapke, Stefan Willmann, Scott-Oliver Grebe, Kristin Menke, Petra A. Thürmann, Sven Schmiedl

**Affiliations:** ^1^ Department of Clinical Pharmacology, School of Medicine, Faculty of Health, Witten/Herdecke University, Witten, Germany; ^2^ Bayer AG, Research and Development, Clinical Pharmacometrics, Wuppertal, Germany; ^3^ Medical Clinic 1, Division of Nephrology, Helios University Hospital Wuppertal, Wuppertal, Germany; ^4^ Bayer AG, Research and Development, Systems Pharmacology and Medicine I, Leverkusen, Germany; ^5^ Philipp Klee-Institute for Clinical Pharmacology, Helios University Hospital Wuppertal, Wuppertal, Germany

**Keywords:** cyclosporine, pharmacokinetics, therapeutic drug monitoring, PBPK, modeling and simulation

## Abstract

This study compared simulations of a physiologically based pharmacokinetic (PBPK) model implemented for cyclosporine with drug levels from therapeutic drug monitoring to evaluate the predictive performance of a PBPK model in a clinical population. Based on a literature search model parameters were determined. After calibrating the model using the pharmacokinetic profiles of healthy volunteers, 356 cyclosporine trough levels of 32 renal transplant outpatients were predicted based on their biometric parameters. Model performance was assessed by calculating absolute and relative deviations of predicted and observed trough levels. The median absolute deviation was 6 ng/ml (interquartile range: 30 to 31 ng/ml, minimum = −379 ng/ml, maximum = 139 ng/ml). 86% of predicted cyclosporine trough levels deviated less than twofold from observed values. The high intra-individual variability of observed cyclosporine levels was not fully covered by the PBPK model. Perspectively, consideration of clinical and additional patient-related factors may improve the model’s performance. In summary, the current study has shown that PBPK modeling may offer valuable contributions for pharmacokinetic research in clinical drug therapy.

## Introduction

Physiologically based pharmacokinetic (PBPK) models are mathematical representations of pharmacokinetic processes. In a bottom-up approach all determining physico-chemical and biological interactions between a drug and the body are implemented into virtual compartments, mapping the anatomical architecture and the physiological and biochemical properties of the body. Integration over time then allows for calculation of concentration-time curves of a modeled drug in specific body-compartments *in silico* (Kuepfer et al., 2016). While PBPK modeling has become an established methodical approach in drug development and latterly in regulatory decision making (Zhao et al., 2012), there is now an emerging use of PBPK modeling to simulate pathological conditions (Radke et al., 2016). Still, the predictive performance of PBPK models in specific clinical settings with heterogeneous and chronically ill patients characterized by numerous unknown individual and clinical factors needs to be further tested.

Cyclosporine is a drug with a high inter- and intraindividual pharmacokinetic variability and a narrow therapeutic window. As a cyclic oligopeptide (Rüegger et al., 1976) cyclosporine shows a poor water-solubility but high intestinal permeability (Amidon et al., 1995). It is predominantly a substrate of cytochrome P450 (CYP) 3A4 (Kronbach et al., 1988) and P-glycoprotein (P-GP) (Saeki et al., 1993). Due to a high protein binding predominantly to lipoproteins (Lemaire and Tillement, 1982), the fraction unbound in blood (f_U_) is low (1–17%) and depends on measurement methods and examined individuals (Akhlaghi and Trull, 2002). Cyclosporine shows saturable accumulation in blood cells and peripheral tissue resulting in a partially non-linear pharmacokinetic behavior (Foxwell et al., 1988; Tanaka et al., 2000). Moreover it shows a high affinity for fatty and lymphatic tissue (Kahan et al., 1983). Cyclosporine is eliminated almost exclusively in form of its metabolites *via* bile (Venkataramanan et al., 1985).

Since the early 1980’s cyclosporine has been used for immunosuppression after renal transplantation and can impressively reduce acute rejection episodes early after transplantation. Yet long-term allograft survival is still inadequate with a progressively decreasing allograft function occurring in most patients within 10 years after transplantation (Nankivell et al., 2003). Causes of so called *chronic-allograft-injury* include, among others, cyclosporine nephrotoxicity and immunological rejection of the transplant which should both be prevented by an optimal immunosuppressive regime. Although dosing and target levels are poorly researched (KDIGO Clinical Practice Guideline for the Kidney Disease Improving Global Outcomes, 2009) and must be adjusted in consideration of individual patient factors and (immunosuppressive) co-medication, a high variability of cyclosporine exposure might correlate with a worse long-term outcome (Kahan et al., 2000). In clinical practice cyclosporine blood levels are measured regularly and invasively *via* therapeutic drug monitoring (TDM) to avoid side effects, particularly nephrotoxicity, as well as rejection episodes and to adapt dosing. However, details on procedure and the actual benefit of TDM for efficacy and safety of cyclosporine therapy have been studied insufficiently (KDIGO Clinical Practice Guideline for the Kidney Disease Improving Global Outcomes, 2009).

PBPK modeling can serve to further investigate the complex pharmacokinetic of cyclosporine. In the 1990’s there have been several approaches to investigate the non-linear pharmacokinetic behavior of cyclosporine after intravenous application by PBPK modeling (Kawai et al., 1998; Tanaka et al., 1999). One model has been applied to analyze the intravenous pharmacokinetic of cyclosporine in children (Gérard et al., 2010). Attempts to describe the oral absorption of cyclosporine by whole-body PBPK modeling either publish incomplete data (Darwich et al., 2013) or rely on semi-mechanistic approaches only (Gertz et al., 2013). Drug levels from TDM allow to retrospectively evaluate the predictive ability of a PBPK model in clinical care (Polasek et al., 2018).

The aim of this study was to implement a PBPK model for the oral application of cyclosporine and to assess its predictive performance in a clinical setting by comparing model based predictions of trough levels with observed trough levels from TDM of renal transplant outpatients.

## Materials and Methods

### Development of the Physiologically Based Pharmacokinetic Model

The PBPK model was constructed using PK-Sim® (Open Systems Pharmacology Suite/Leverkusen, Germany). The PK-Sim® workflow, basic algorithm, and differential equations have been detailed previously (Willmann et al., 2003; Willmann et al., 2005). For the identification and quantification of model parameters a comprehensive literature search was conducted. For parameters with a broad range of values or no values found in literature a sensitivity analysis was performed. Finally applied values were estimated by comparing model based predictions with the pharmacokinetic data of single dose healthy volunteer studies published in literature (Table 1). A clinically and pharmacokinetically relevant range of doses was taken into account (for intravenous application: 1.5 mg/kg body weight (BW), 2, 2.1, 2.5, 3.0, and 5.0 mg/kg BW, for oral application: 100, 300, and 600 mg). For the simulations a virtual individual with the biometric values of an average European male human was generated (age: 30 years, BW: 73 kg, body height (BH): 176 cm). Model performance was assessed visually and quantitatively comparing the predicted and observed concentration-time curves in venous blood (Table 2).

**TABLE 1:** Single dose healthy volunteer studies used to evaluate the developed PBPK model.

Reference Min et al. (2000)	
Number of volunteers^a^	11
Intravenous dose, mg/kg body weight	1.5
Infusion time, h	3
Age, years [mean (+/− SD)]	28.6 (±5.7)
Body weight, kg [mean (+/− SD)]	74.3 (±12.0)
Body height, cm	*not published*
Sex, f/m	4/7
Reference Gomez et al. (1995)	
Number of volunteers^a^	1
Intravenous dose, mg/kg body weight	2
Infusion time, h	2.5
Age, years[mean (+/− SD)]	*not published*
Body weight, kg [mean (+/− SD)]	*not published*
Body height, cm	*not published*
Sex, f/m	1/0
Reference Ptachcinski et al. (1987)	
Number of volunteers^a^	1
Intravenous dose, mg/kg body weight	2.1
Infusion time, h	2
Age, years (mean (+/− SD))	*not published*
Body weight, kg (mean (+/− SD))	*not published*
Body height, cm	*not published*
Sex, f/m	*not published*
Reference Ducharme et al. (1995)	
Number of volunteers^a^	10
Intravenous dose, mg/kg body weight	2.5
Infusion time, h	3
Age, years [mean (+/− SD)]	27.6 (±6.5)
Body weight, kg [mean (+/− SD)]	76 (±9.2)
Body height, cm	*not published*
Sex, f/m	0/10
Reference Hebert et al. (1992)	
Number of volunteers^a^	1
Intravenous dose, mg/kg body weight	3
Infusion time, h	2.5
Age, years (mean (+/− SD))	*not published*
Body weight, kg (mean (+/− SD))	*not published*
Body height, cm	*not published*
Sex, f/m	0/1
Reference Ehinger et al. (2013)	
Number of volunteers^a^	52
Intravenous dose, mg/kg body weight	5
Infusion time, h	4
Age, years [mean (range)]	24.4 (18–46)
Body weight, kg [mean (range)]	70.4 (60–99.8)
Body height, cm	*not published*
Sex, f/m	19/33
Reference Kees et al. (2006)	
Number of volunteers^a^	6
Oral dose, mg	100
Age, years [mean (range)]	25 (22–29)
Body weight, kg [mean (range)]	72 (63–90)
Body height, cm	180 (171–192)
Sex, f/m	0/6
Reference Kees et al. (2006)	
Number of volunteers^a^	6
Oral dose, mg	300
Age, years [mean (range)]	25 (22–29)
Body weight, kg [mean (range)]	72 (63–90)
Body height, cm	180 (171–192)
Sex, f/m	0/6
Reference Kees et al. (2006)	
Number of volunteers^a^	6
Oral dose, mg	600
Age, years [mean (range)]	25 (22–29)
Body weight, kg [mean (range)]	72 (63–90)
Body height, cm	180 (171–192)
Sex, f/m	0/6

SD, standard deviation; f, female. m, male.

a Number of volunteers represented by the published concentration-time curve.

**TABLE 2:** Evaluation of the developed PBPK model with single dose healthy volunteer studies (Table 1).

Intravenous dose 1.5 mg/kg body weight Min et al. (2000)				
	Predicted (n = 1)		Observed (n = 11)	Prediction fold-difference
AUC_T_END_, ng*h/ml	6,415		6,523 (mean)	0.98
C_MAX_, ng/ml	1,369		1,399 (mean)	0.98
t_MAX_, h	3.0		3.04 (mean)	0.99
**Intravenous dose 2 mg/kg body weight** Gomez et al. (1995)				
	**Predicted (n = 1)**		**Observed (n = 1)**	**Prediction fold-difference**
AUC_T_END_, ng*h/ml	9,466		8,923	1.06
C_MAX_, ng/ml	2,193		2096	1.05
t_MAX_, h	2.5		2.48	1.01
**Intravenous dose 2.1 mg/kg body weight** Ptachcinski et al. (1987)				
		**Predicted (n = 1)**	**Observed (n = 1)**	**Prediction fold-difference**
AUC_T_END_, ng*h/ml		10.145	7,492	1.35
C_MAX_, ng/ml		2,703	2,105	1.28
t_MAX_, h		3	3.04	0.99
**Intravenous dose 2.5 mg/kg body weight** Ducharme et al. (1995)				
		**Predicted (n = 1)**	**Observed (n = 10)**	**Prediction fold-difference**
AUC_T_END_, ng*h/ml		12.504	10.003	1.25
C_MAX_, ng/ml		2,600	2,161	1.20
t_MAX_, h		3	2.01	1.49
**Intravenous dose 3 mg/kg body weight** Hebert et al. (1992)				
		**Predicted (n = 1)**	**Observed (n = 1)**	**Prediction fold-difference**
AUC_T_END_, ng*h/ml		15.834	11.878	1.33
C_MAX_, ng/ml		3,725	3,027	1.23
t_MAX_, h		2.5	3.04	0.82
**Intravenous dose 5 mg/kg body weight** Ehinger et al. (2013)				
		**Predicted (n = 1)**	**Observed (n = 52)**	**Prediction fold-difference**
AUC_T_END_, ng*h/ml		29.157	20.778	1.40
C_MAX_, ng/ml		5,700	3,071	1.86
t_MAX_, h		4	4.44	0.90
**Oral dose 100 mg** Kees et al. (2006)				
		**Predicted (n = 1)**	**Observed (n = 6)**	**Prediction fold-difference**
AUC_T_END_, ng*h/ml		1,625	1,553	1.05
C_MAX_, ng/ml		516	511	1.01
t_MAX_, h		1.25	1.5	0.83
F_A_		0.98	0.9 (Gertz et al., 2013)	1.09
F_I_		0.41	0.47 (Wu et al., 1995)	0.87
F		0.27	0.3 (Akhlaghi and Trull, 2002)	0.9
**Oral dose 300 mg** Kees et al. (2006)				
		**Predicted (n = 1)**	**Observed (n = 6)**	**Prediction fold-difference**
AUC_T_END_, ng*h/ml		6,432	5,050	1.27
C_MAX_, ng/ml		1,578	1,277	1.24
t_MAX_, h		1.45	1.51	0.96
F_A_		0.97	0.9 (Gertz et al., 2013)	1.08
F_I_		0.43	0.47 (Wu et al., 1995)	0.91
F		0.28	0.3 (Akhlaghi and Trull, 2002)	0.93
**Oral dose 600 mg** Kees et al. (2006)				
		**Predicted (n = 1)**	**Observed (n = 6)**	**Prediction fold-difference**
AUC_T_END_, ng*h/ml		10.693	9,630	1.11
C_MAX_, ng/ml		2,269	1919	1.18
t_MAX_, h		1.85	2.5	0.74
F_A_		0.74	0.9 (Gertz et al., 2013)	0.82
F_I_		0.57	0.47 (Wu et al., 1995)	1.21
F		0.28	0.3 (Akhlaghi and Trull, 2002)	0.93

AUC_T_END_, area under curve from the start to the end of the simulation; C_MAX_, maximum concentration; t_MAX_, time at which the maximum concentration is assumed; F_A_, fraction of the drug dose absorbed into and through the gastrointestinal membranes; F_I_, fraction of the absorbed dose that passes through the gut into the hepatic portal blood unmetabolized; F, absolute oral bioavailability.

Modeling and evaluation were initially performed for the intravenous application of cyclosporine. In a second step the oral absorption process was added. For modeling of the absorption process the fraction of the drug’s dose absorbed into and through the gastrointestinal membranes [F_A_ = 0.9 for cyclosporine (Gertz et al., 2013)], the fraction of the absorbed dose that passes through the gut into the hepatic portal blood without metabolization [F_I_ = 0.47 for cyclosporine (Wu et al., 1995)] and the absolute oral bioavailability [F = 0.3 for cyclosporine (Akhlaghi and Trull, 2002)] were considered while defining the model parameter’s values. That is, solubility, specific intestinal permeability, and intestinal intrinsic clearance were fitted within a range of literature based values in order to meet literature based values for F_A_, F_I_, and F.

### Model Parameters

Table 3 shows all cyclosporine-specific model parameters and their respective values employed within the model as well as the abundance of binding proteins, transporters and metabolizing enzymes utilized to further characterize binding, transport and biotransformation processes in the virtual individuals.

**TABLE 3:** PBPK model input parameters.

Physicochemical	
Molecular weight, g/mol	1,203 (Rüegger et al., 1976)
pKa value	Neutral (Rüegger et al., 1976)
Lipophilicity (logP), log units	3.25
Solubility, µg/ml	190
Specific intestinal permeability, cm/min	4.5^−5^
Binding	
Fraction unbound in plasma	0.06 (Tanaka et al., 1999)
Specific binding to pulmonary tissue	K_M_ = 0.05 μmol/L (Tanaka et al., 1999)
	K_OFF_ = 4.75/s (Tanaka et al., 1999)
	B_C_ = 8.48 μmol/L (Tanaka et al., 1999)
Specific binding to cardiac tissue	K_M_ = 0.02 μmol/L (Tanaka et al., 1999)
	K_OFF_ = 2.18/s (Tanaka et al., 1999)
	B_C_ = 3.72 μmol/L (Tanaka et al., 1999)
Specific binding to bone tissue	K_M_ = 0.28 μmol/L (Tanaka et al., 1999)
	K_OFF_ = 27.68/s (Tanaka et al., 1999)
	B_C_ = 18.7 μmol/L (Tanaka et al., 1999)
Specific binding to dermal tissue	K_M_ = 0.27 μmol/L (Tanaka et al., 1999)
	K_OFF_ = 27.18/s (Tanaka et al., 1999)
	B_C_ = 25.9 μmol/L (Tanaka et al., 1999)
Specific binding to renal tissue	K_M_ = 0.5 μmol/L (Tanaka et al., 1999)
	K_OFF_ = 0.00389/h (Tanaka et al., 1999)
	B_C_ = 104 μmol/L (Tanaka et al., 1999)
Specific binding to splenic tissue	K_M_ = 0.56 μmol/L (Tanaka et al., 1999)
	K_OFF_ = 0.00225/h (Tanaka et al., 1999)
	B_C_ = 132 μmol/L (Tanaka et al., 1999)
Specific binding to hepatic tissue	K_M_ = 0.15 μmol/L (Tanaka et al., 1999)
	K_OFF_ = 0.00271/h (Tanaka et al., 1999)
	B_C_ = 38.7 μmol/L (Tanaka et al., 1999)
Specific binding to intestinal tissue	K_M_ = 0.74 μmol/L (Tanaka et al., 1999)
	K_OFF_ = 0.00558/h (Tanaka et al., 1999)
	B_C_ = 78.1 μmol/L (Tanaka et al., 1999)
Specific binding to blood cells	K_M_ = 0.15 μmol/L (Tanaka et al., 1999)
	K_OFF_ = 150/s (Tanaka et al., 1999)
	B_C_ = 3.86 μmol/L (Tanaka et al., 1999)
Efflux transport	
Specific efflux transport in brain	K_M_ = 0.09 μmol/L (Tanaka et al., 1999)
	V_MAX_ = 2.14 nmol/ml/min (Tanaka et al., 1999)
	B_C_ = 1 μmol/L (Tanaka et al., 1999)
Specific efflux transport in intestine	K_M_ = 0.09 μmol/L (Tanaka et al., 1999)
	V_MAX_ = 2.14 nmol/ml/min (Tanaka et al., 1999)
	B_C_ = 1 μmol/L for colon and a relative distribution aboral of 0.55 for ileum, 0.38 for jejunum, and 0.07 for duodenum (Bruyère et al., 2010)
Metabolism	
Systemic CYP3A4 biotransformation	K_M_ = 0.5 nmol/ml (Tanaka et al., 1999)
	V_MAX_ = 0.78 nmol/min/g tissue (Tanaka et al., 1999)
	B_C_ = 4.32 μmol/L for liver (Rodrigues, 1999) and a relative distribution of 0.71 for large intestine (non-mucosal tissue), 0.4 for small intestine (non-mucosal tissue), 0.35 for stomach, 0.03 for kidney, 0.01 for muscle and brain (Bayer Technology Services, 2012)
Intestinal CYP3A4 biotransformation	Cl_II_ = 50 L/h
	B_C_ = 1.08 μmol/L for duodenum (Wagner et al., 2013)
	B_C_ = 1.05 μmol/L for upper jejunum (Wagner et al., 2013)
	B_C_ = 0.99 μmol/L for lower jejunum (Wagner et al., 2013)
	B_C_ = 0.84 μmol/L for upper ileum (Wagner et al., 2013)
	B_C_ = 1.44 μmol/L for lower ileum (Wagner et al., 2013)

If no reference is given, the particular parameter value was approached by fitting the PBPK model to observed data. K_M_, Michaelis-Menten constant (substrate concentration at half-maximum reaction rate); V_MAX_, maximum reaction rate; K_OFF_, dissociation constant; B_C_, concentration of binding/metabolizing protein; Cl_II_, intestinal intrinsic clearance.

For the lipophilicity and the solubility of cyclosporine depending on the solvent a broad range of values was found in literature. Applied values were within the range of literature based values and were adapted to finally map literature based concentration-time curves of healthy individuals and a literature based value for F_A_, respectively, as described above.

Nine specific binding processes to parenchymatous organs and blood cells were defined and quantified according to PBPK modeling for intravenous application of cyclosporine by Kawai and Tanaka et al. (Kawai et al., 1998; Tanaka et al., 1999) that were based on invasive studies with rats (Tanaka et al., 2000). From the same literature source the quantification of f_U_ was set.

The clearance of cyclosporine from the body was represented *via* CYP3A4 enzymatic activity in the liver, the stomach, the kidney, the muscles and the intestine. The kinetics of systemic biotransformation were again quantified based on Tanaka et al. (Tanaka et al., 1999). While this model was developed for intravenous application and supposed biotransformation in the liver only, a closer agreement with observed data could be achieved with the inclusion of biotransformation in more CYP 3A4-enriched organs as mentioned above. The abundance and relative distribution of CYP 3A4 was quantified based on *in vitro* data as specified in Table 3.

To reproduce the bioavailability of cyclosporine after oral intake the intestinal biotransformation during absorption was quantified excluding systemic metabolism by fitting the intrinsic intestinal clearance to reach a literature based value for F_I_.

Ancillary, a P-GP-efflux-transport for the blood-brain-barrier and the intestinal wall was defined. The quantification of the efflux kinetics relied on Tanaka et al. (Tanaka et al., 1999) while the absolute and relative protein abundance for the intestine and brain was set in agreement with observed and *in vitro* data, respectively.

Since only a negligible amount of cyclosporine is cleared without metabolization and TDM in blood was supposed to be specific, no excretion processes were implemented within the model. Simulations with cyclosporine-intake as a solution (i.e. no drug liberation) showed the best agreement with observed data.

### Drug Levels From Therapeutic Drug Monitoring

Available drug levels from TDM consisted of 356 cyclosporine trough levels of 32 renal transplant outpatients [male/female: 16/16, median age: 47 years (range: 22–66 years), median time after transplantation: 4 years (range: 1–20 years)] attended at the nephrological outpatient clinic of university hospital Marburg, Germany. Patients received a maintenance immunosuppressive medication regime consisting of cyclosporine (median drug dose: 87.5 mg every 12 h, range: 25–225 mg), additionally mycophenolate mofetil or azathioprine and, if necessary, corticosteroids. All patients showed a chronic elevation of serum creatinine levels and/or proteinuria. TDM was conducted over 6 years with an interval of one month or longer between each sample. Due to individual dose adjustments over time, each patient had received between 1 and 11 different doses of cyclosporine (median: 2) during the observation period. Cyclosporine levels were determined as whole blood measurements.

### Comparison of Model Based Predictions With Drug Levels From Therapeutic Drug Monitoring

To compare clinically observed cyclosporine trough levels (C0_obs) with predictions made by the PBPK model (C0_pred) for each patient a corresponding virtual patient was created. Patient-specific parameters taken therefore into account were sex, age (as the median value of the observation period) and BW (as the median value of the observation period) (Table 4). For BH no values were documented in the retrospectively used clinical data. After assessing the resulting bias as negligible (data not shown) the parameter BH was quantified with the German average in correspondence to age and sex (Statistisches Bundesamt, 2013).

**TABLE 4:** Biometric parameters used to characterize each clinical patient virtually for modeling and simulation.

Patient ID	Sex	Age, years	Body weight, kg	Body height, m
1	F	64	71	1.64
2	F	59	68	1.65
3	M	48	111	1.80
4	M	66	86	1.76
5	M	22	106	1.81
6	f	48	64	1.67
7	f	59	80	1.65
8	m	58	103	1.78
9	f	36	48	1.67
10	f	49	63	1.67
11	f	41	53	1.67
12	m	50	94	1.79
13	f	49	69	1.67
14	f	24	106	1.68
15	f	62	55	1.64
16	f	51	74	1.66
17	m	36	99	1.80
18	m	30	75	1.80
19	m	57	76	1.78
20	f	54	53	1.66
21	m	27	66	1.81
22	m	42	78	1.80
23	m	46	93	1.80
24	m	37	77	1.80
25	f	43	64	1.67
26	f	41	59	1.67
27	f	44	62	1.67
28	m	41	77	1.80
29	f	45	76	1.67
30	m	57	95	1.78
31	m	42	63	1.80
32	m	52	81	1.79

ID, identification. m, male; f, female; Sex, age and body weight rely on individual data of the clinical population; Presumable body height was set with the German average with respect to sex and age as publicated by the German federal statistical office (Statistisches Bundesamt, 2013).

Trough levels were predicted for each patient and each dose with an application scheme of administrating the corresponding dose every 12 h over a period of 4 days to reach steady state.

Comparison was conducted by calculating the absolute deviation

(C0_pred−C0_obs)

and ratio

(C0_pred/C0_obs)

for matched pairs of trough levels. For the ratio a scatter-plot was mapped and the residuum R

R=log(C_pred/C_obs)

was calculated, where a value of −0.30 < R < 0.30 represents a deviation of C0_pred and C0_obs of less than factor two and a value of −0.48 < R < 0.48 represents a deviation of C0_pred and C0_obs of less than factor three. The residuum of each matched pair of trough levels was then stratified for BW normalized dose and patient to reveal a potential relationship between prediction error and dose or prediction error and the specific patient, respectively.

To further characterize the predictive performance of the PBPK model the bias B

 B = Mean (C_obs - C_pred)

the precision *p*

P=Root(Mean (C_obs - C_pred)^2))

the mean relative deviation MRD

MRD = 10^x,x=Root (Mean ((log C_obs - ⁡log C_pred)^2)) 

and the mean percentage error MPE

MPE = Mean((C_pred - C_obs)/C_obs ∗ 100) 

were calculated (Sheiner and Beal, 1981; Edginton et al., 2006; Khalil and Läer, 2014).

Numerical calculations and graphical illustrations were conducted using Microsoft Excel® (Washington, United States of America).

## Results

### Evaluation of the Developed Physiologically Based Pharmacokinetic Model Using Single Dose Healthy Volunteer Studies

Results of the quantitative comparison of predicted and observed concentration-time curves with respect to F_A_, F_I_ and F for healthy individuals represented by AUC_T_END_, C_MAX_, and t_MAX_ are shown in Table 2. Particularly for low doses a high accordance could be achieved. For higher doses the predicted values showed a trend to exceed the observed values. For high oral dosing the model showed a solubility based restriction of absorption indicated by a fall of F_A_. This led to a raise of F_I_ resulting in a constant value of absolute oral bioavailability (F).

### Comparison of Model Based Predictions With Drug Levels From Therapeutic Drug Monitoring

**FIGURE 1:**
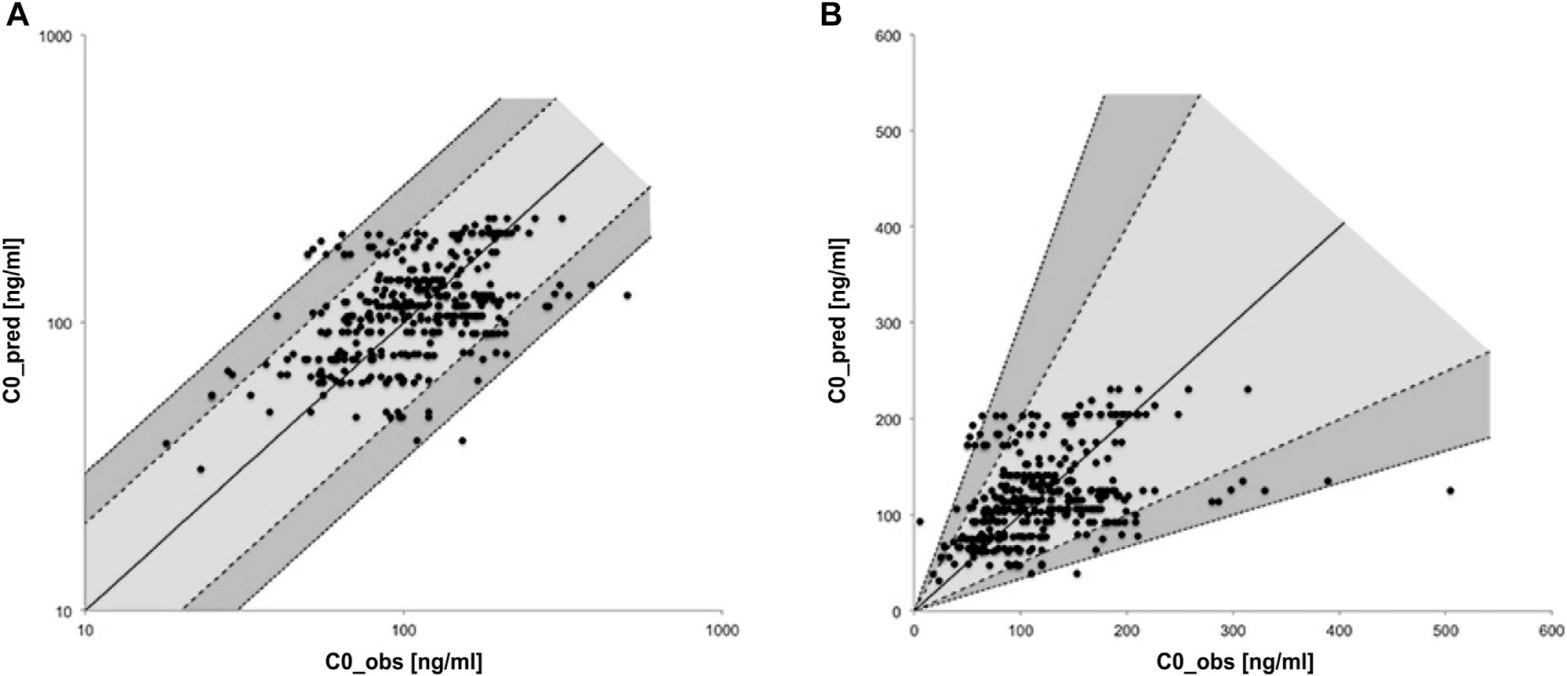
Scatter-plot of relative deviation between predicted and observed trough levels in logarithmic **(A)** and linear **(B)** scale.

C0_pred for renal transplant patients was 114 ng/ml in the median [interquartile range (IQR): 84–141 ng/ml, minimum (MIN) = 31 ng/ml, maximum (MAX) = 231 ng/ml] while C0_obs came to 111 ng/ml (IQR: 79–152 ng/ml, MIN = 5 ng/ml, MAX = 504 ng/ml). Comparing each corresponding pair of specific patient and dose the absolute deviation of C0_pred and C0_obs was 6 ng/ml in the median (IQR: −30–31 ng/ml, MIN = −379 ng/ml, MAX = 139 ng/ml). The ratios of C0_pred and C0_obs are shown as a scatterplot in Figure 1 in logarithmic and linear scale. 307 matched pairs (86%) are located on the light gray area between the dashed lines and differ thus less than twofold. 40 pairs (11%) are located on the dark gray area between dashed and dotted lines and thus differ at least twofold but less than threefold. 9 pairs (3%) exhibited a threefold or even higher deviation as indicated by location beyond the gray areas.

**FIGURE 2:**
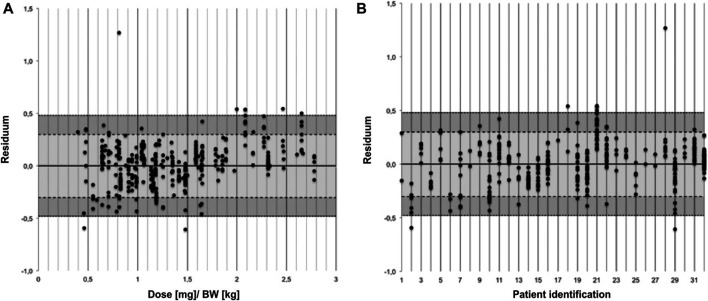
Residua of predicted and observed trough levels stratified by body weight normalized dose **(A)** and by patient **(B)**.

Figure 2 shows the relative deviation of predicted and observed cyclosporine levels represented as the residual and stratified by BW-normalized dose and by patient, respectively. Stratifying the residua by BW-normalized dose revealed a trend toward increasingly positive residua for higher BW-normalized doses, that is the PBPK model predicted higher drug levels for higher doses than clinically observed.

For some patients (for example patient ID numbers 2 and 21) deviations between C0_pred and C0_obs were scattered around a high absolute residuum while predictions for other patients seemed to be scattered around a residuum of 0 (for example patient number 16). Yet others spread around a residuum of 0 and deviated only meanwhile in the direction of a higher residuum (for example patient number 29). The apparent outlier (R = 1.27 for patient number 28) resulted from a trough level of a 41 year old patient with a BW of 77 kg 19 years after transplantation. C0_obs was 5 ng/ml for a dose of 62.5 mg while C0_pred was 93 ng/ml.

The developed PBPK model showed a slightly negative systematic bias (−0.9 ng/ml) and a small standard deviation (precision 58.3 ng/ml). The mean relative deviation was 1.6 and the mean percentage error was 19.3%.

## Discussion

The aim of this study was to test the predictive performance of a PBPK model in real-life clinical care by comparing predicted trough levels of a PBPK model developed for orally administered cyclosporine with observed trough levels measured by TDM in renal transplant outpatients. Up to now, PBPK models are hardly applied in clinical routine. Our work has shown, that taking into account patient's biometric parameter a PBPK model shows a satisfying accuracy with a small systematic bias and a reasonable precision for a heterogenous and chronically ill group of patients.

While in most patients predicted trough levels were in good accordance with observed drug levels, a relevant deviation (more than threefold) was found in some patients at least for a few cyclosporine trough levels. Prediction errors were more pronounced in patients receiving higher cyclosporine doses showing an overprediction in most of these cases. In the literature, factors influencing the absorption process are discussed as being substantial for the pharmacokinetic variability of cyclosporine. These factors include diet, intestinal motility and notably factors with an impact on the intestinal cytochrome P450 system.

Comedications inducing or inhibiting CYP3A4 have a strong impact on cyclosporine’s pharmacokinetics by lowering plasma levels up to one third or doubling of oral bioavailability, respectively (Hebert et al., 1992; Gomez et al., 1995). In our study, documented comedication data were limited to a few concomitantly taken immunosuppressive compounds (azathioprine, mycophenolate mofetil, and corticosteroids) which might be considered as a potential source for bias. Whereas conflicting results have been reported for corticosteroids, no clinically relevant influence on cyclosporine pharmacokinetics have been reported for azathioprine and mycofenolate mofetil (Kuypers, 2008; Lam et al., 2008). Hence, a limited impact of the immunosuppressive agents taken by the patients on the cyclosporine pharmacokinetics cannot be fully excluded. However, we did not found relevant discrepancies regarding the predictive accuracy of the developed cyclosporine PBPK model after stratification by corticosteroid intake (data not shown). Due to the limited documentation of comedications, we cannot fully exclude an intake of other comedications influencing the pharmacokinetics of cyclosporine to a relevant extent. Inclusion of those interacting (non-) immunosuppressive comedications may further improve the predictive accuracy of cyclosporine PBPK models.

It is known that expression of CYP3A4 can change in chronic kidney disease (Rowland Yeo et al., 2011; Zhao et al., 2012) which mostly develops in the clinical course after kidney transplantation and was present in our clinical population too. We stratified the model’s prediction by patient’s GFR (data not shown) and found no correlation between GFR and accuracy of model prediction. This is in accordance with the available evidence. Correspondingly no dose adjustments are recommended for patients with impaired kidney function (Novartis Pharma, 2011) implicating that changes of CYP3A4 expression in chronic kidney disease are of minor pharmacokinetic relevance for cyclosporine. Still, modeling of cyclosporine metabolism in chronic kidney disease seems to be an interesting spot for further research. The representation of intestinal cyclosporine metabolism within the model is based on literature based local CYP3A4 expression and on an intrinsic clearance fitted to match a literature based value for F_I_ of 0.47. Other attempts to derive the intestinal extraction rate for cyclosporine from *in vitro*-data by PBPK modeling resulted in a 5-fold underestimation of the supposed value for F_I_ (Gertz et al., 2011). Considerable variations in local expression influencing clearance *in vivo* are well known for CYP3A4 (Paine et al., 1997). Moreover, for cyclosporine its binding not only to plasmatic but also to cytosolic lipoproteins could be of particular relevance for intestinal and systemic metabolism. Lipoproteins are involved in many highly regulated physiological and pathophysiological processes which might also concern lipoprotein-bound cyclosporine (Gupta and Benet, 1990). Distribution of cyclosporine into lipoproteins shows an extensive inter- and intraindividual variation (Sgoutas et al., 1986) and lipoproteins vary greatly according to the current metabolic state in general, during cyclosporine therapy and with concurrent chronic kidney disease in particular. Depending on the method for measurement, pre-analytics and examined individuals values for f_u_ vary therefore from 1 to 17% (Akhlaghi and Trull, 2002) while the fraction unbound was a very sensitive parameter in the current modeling with a distinct influence on simulated concentration-time curves. Concerning this aspect the developed PBPK model might not cover all physiological and pathophysiological conditions relevant for a mechanistic representation and mapping of cyclosporine pharmacokinetic variability but might offer prospects for further research.

Intestinal P-GP is discussed as another potential cause for the high variability of cyclosporine absorption (Fricker et al., 1996; Lown et al., 1997). The quantification of the intestinal P-GP efflux transport kinetics in the current model is based on the efflux transport of cyclosporine at the blood-brain-barrier of rats *in vivo* (Tanaka et al., 1999) which is attributed to P-GP (Goralski et al., 2006). Using numerical values for the P-GP efflux transport kinetics from *in vitro* studies (Saeki et al., 1993; Fricker et al., 1996) resulted in a fraction absorbed of ∼0.01, i.e. almost no absorption. Difficulties in translating P-GP *in vitro* data to *in vivo* observations are well known and might be at least partially caused by the complex interplay of intestinal solubility, intestinal permeability and intestinal metabolism. Modeling and simulation in the current work confirmed the assumption that a P-GP-efflux-transport in the small intestinum might be of minor importance for the pharmacokinetic profile of cyclosporine since a large amount of drug must be absorped to enable the extensive intestinal metabolism known for cyclosporine. P-GP might be of particular relevance to prevent absorption from the large intestine thereby forming an absorption window for cyclosporine in jejunum and ileum (Fricker et al., 1996). Moreover, a relevance of P-GP-efflux for the systemic distribution of cyclosporine was discussed before (Schinkel et al., 1995), but not considered in the current model.

The intravenous pharmacokinetics of cyclosporine within the model are marked by nine specific binding processes in blood cells and in parenchymatous organs leading to the high volume of distribution and a long half-life. While the accumulation of cyclosporine in blood cells due to specific and high-affinity-binding to cyclophilins is well studied, binding of cyclosporine in peripheral tissue and its quantification was hypothesized based on tissue concentrations in rates and estimations made by PBPK-modeling (Tanaka et al., 1999; Tanaka et al., 2000). An actual physiological correlate is unknown or at least presumably nonspecific (Ryffel, 1993). The validity of an interspecies translation between rats and humans was not studied.

Rather than specific binding processes, biotransformation and specific transports, physico-chemical parameters, that is solubility and intestinal permeability, appeared to be very sensitive parameters having a strong impact on predicted concentration-time curves and trough levels in the developed PBPK model and the conducted simulations by influencing absorption. Depending on the solvent, values for cyclosporine solubility range from 7.3 μg/ml in water (Ismailos et al., 1991) up to 250 μg/ml in postprandial human intestinal fluid (Persson et al., 2005). A solubility of 190 μg/ml as used in the current work seems to be a reasonable parameter value resulting in a solubility limit and a decline of the fraction absorbed at doses above 300 mg per os. This is in accordance with observed data and the reduction of dose has been discussed as a possible reason for the increase of oral bioavailability that can be observed in the course of cyclosporine immunosuppressive therapy early after transplantation (Ptachcinski et al., 1986). With respect to the fact that the absorption of cyclosporine seems to be food-dependent (Gupta and Benet, 1990), enhanced mechanisms of digestion prior to absorption might be involved in the absorption process. The prediction of drug levels while considering food intake and adapting solubility in the PBPK model might help to map very low and very high drug levels.

To the best of our knowledge, two other whole-body PBPK models depicting the absorption process of cyclosporine can be found in the literature. The first model (Darwich et al., 2013) relies on the Simcyp® compound file for cyclosporine, available in the Simcyp® Simulator compound library. Only few model parameter values are reported within the publication itself. F_I_ is supposed to be much higher and F_A_ to be considerably smaller than in the current work but no references for these assumptions are published. In the second model (Gertz et al., 2013) a drug liberation process is implied and the intestinal permeability is set higher than in our model. No P-GP kinetics are considered and the intestinal metabolism is represented semi-mechanistically only.

Considering solubility, intestinal metabolism and P-GP-efflux transport mechanistically our model can be used to examine the influence of clinical aspects on cyclosporine dosing (such as non-immunosuppressive co-medication, nutrition, dyslipidemia and chronic kidney disease). This might help to further understand inter- and intraindividual variability and to improve clinical outcome. While drug levels from TDM are a valuable starting point for testing the predictive performance of a PBPK model in a clinical context, further research should evaluate the PBPK model prospectively using full pharmacokinetic profiles.

In summary, the current study has shown that PBPK modeling offers valuable contributions for pharmacokinetic research in clinical populations.

## Data Availability

The raw data supporting the conclusion of this article will be made available by the authors, without undue reservation.

## Abbreviation

PBPK model, physiologically based pharmacokinetic model; CYP, cytochrome P450; P-GP, P-glycoprotein; f_U_, fraction nbound; TDM, therapeutic drug monitoring; BW, body weight; BH, body height; F_A,_ fraction of the drug’s dose absorbed into and through the gastrointestinal membranes; F_I_, fraction of the absorbed dose that passes through the gut into the hepatic portal blood unmetabolized; F, absolute oral bioavailability; C0_obs, clinically observed cyclosporine trough levels; C0_pred, cyclosporine trough levels predicted by the PBPK model; R, residuum; B, bias; P, precision; MRD, mean relative deviation; MPE, mean percentage error; IQR, interquartile range; MIN, minimum; MAX, maximum.
